# Molecular phylogeny of the *Acer*-feeding aphid subfamily Drepanosiphinae (Insecta: Hemiptera: Aphididae) and the evolution of its endosymbiotic consortia

**DOI:** 10.1186/s40851-025-00255-2

**Published:** 2025-12-13

**Authors:** Kamila Malik, Emmanuelle Jousselin, Anne-Laure Clamens, Shun’ichiro Sugimoto, Karina Wieczorek

**Affiliations:** 1 Institute of Biology, Biotechnology and Environmental Protection, Faculty of Natural Sciences, University of Silesia in Katowice, Bankowa, 9, Katowice, 40–007 Poland; 2 UMR 1062 Centre de Biologie pour la Gestion des Populations, INRAe, CIRAD, Institut Agro, IRD, Univ. Montpellier, 755 avenue du Campus Agropolis, Montferrier-sur-Lez, 34988 France; 3 Yokohama Plant Protection Station, 5-57 Kitanakadori, Nakaku, Yokohama, 231-0003 Japan

**Keywords:** Aphid microbiome, *Drepanaphis*, Dual symbiosis, Maple, *Sodalis*, *Yamatocallis*

## Abstract

The Drepanosiphinae is a Holarctic subfamily of Aphididae comprising six genera: *Drepanaphis, Drepanosiphoniella, Drepanosiphum, Megalosiphonaphis, Shenahweum,* and *Yamatocallis*, all of which exhibit strict host plant associations, primarily with *Acer* species. Despite long-standing taxonomic attention, evolutionary relationships within the group remain poorly resolved, and some important aspects of their biology, such as their patterns of association with symbionts, have been unexplored despite evidence that species in the subfamily might be involved in atypical nutritional symbioses. Here, we present a molecular phylogenetic reconstruction of this subfamily and investigate the evolution of its endosymbiotic consortia. Phylogenetic analyses were conducted using multiple DNA markers, employing both Bayesian inference (BI) and maximum likelihood (ML) approaches. Endosymbionts were characterized using high-throughput sequencing of a fragment of the bacterial 16S rRNA gene. The resulting phylogenies are largely congruent across markers and methods and consistently support the monophyly of Drepanosiphinae. *Drepanaphis* and *Drepanosiphum* form a well-supported clade as sister to *Drepanosiphoniella*, while *Yamatocallis* and *Megalosiphonaphis* form a distinct, more distantly related clade. Within *Drepanaphis*, species group according to host plant use rather than traditional morphological groupings, revealing three host-associated clades: *rubrum*, *saccharum*, and *grandidentatum*. Endosymbiont characterization revealed that, in addition to the obligate symbiont *Buchnera aphidicola*, most Drepanosiphinae species also host a *Sodalis*-like bacterium, consistent with previous genomic evidence for a dual nutritional symbiosis with this bacterium. However, *Sodalis* was absent in most *Yamatocallis* species, indicating a complex and potentially dynamic evolutionary history of symbiotic relationships within the subfamily. Patterns of association with *Wolbachia*, *Rickettsia, Fukatsuia, Serratia* and *Arsenophonus* suggest a limited role in nutrition. By integrating phylogenetic reconstruction with symbiont profiling, this study provides the most comprehensive evolutionary framework to date for Drepanosiphinae and reinforces the view that nutritional symbioses in aphids are evolutionarily dynamics.

**Supplementary Information:**

The online version contains supplementary material available at 10.1186/s40851-025-00255-2.

## Background

Drepanosiphinae is a morphologically and biogeographically diverse subfamily within Aphididae [1]. It comprises 42 recognized species within six genera: *Drepanaphis* Del Guercio, 1909 (18 species), *Drepanosiphoniella* Davatchi, Hille Ris Lambers & Remaudière, 1957 (three species), *Drepanosiphum* Koch, 1855 (10 species), *Megalosiphonaphis* Sugimoto, 2024 (one species), *Shenahweum* Hottes & Frison, 1931 (one species), *Yamatocallis* Matsumura, 1917 (nine species) [2, 3]. Drepanosiphinae are distributed across most major biogeographic regions. *Drepanosiphum* is Holarctic and well-represented in Europe, while *Drepanosiphoniella* occurs mainly in the Mediterranean. The Nearctic genera *Shenahweum* and *Drepanaphis* account for much of North American diversity, with *Drepanaphis* being the most speciose; only *D. acerifoliae* (Thomas, 1878) occurs outside North America, also reaching Europe and Japan. In Asia, *Yamatocallis* occurs in India, China, Korea, and Japan, whereas *Megalosiphonaphis* has only been recorded in Japan [3–11]. Across all regions, these aphids exhibit strong host specificity, primarily associating with *Acer* species [3]. Morphologically, representatives of Drepanosiphinae are distinguished by features such as enlarged fore or mid femora and the presence of rastral spines on the hind tibiae, which are considered synapomorphies for the group [1]. All genera within the subfamily are morphologically well characterized and have been the subject of detailed taxonomic studies [1, 4, 6, 8, 9, 11, 12].

Despite this, evolutionary relationships within Drepanosiphinae remain poorly resolved due to limited taxon sampling [1]. In particular, high morphological similarity among species in the largest genus, *Drepanaphis*, has long complicated species delimitation [13]. To address this, five morpho-groups have been proposed based on diagnostic traits such as the shape and color of the pterostigma, the form of the siphunculi, and the presence and structure of abdominal tubercles [9, 13]. However, several species remain difficult to place confidently within these morpho-groups, suggesting that morphology alone may be insufficient for accurate species delimitation. Furthermore, the phylogenetic position of the Asiatic genera *Yamatocallis* and *Megalosiphonaphis* remain unresolved, emphasizing the need for broader molecular phylogenetic analyses.

Thus, a comprehensive molecular phylogeny is needed to resolve evolutionary relationships within Drepanosiphinae, clarify the placement of Asiatic genera and test the validity of *Drepanaphis* morpho-groups. Given the strong host specificity toward *Acer* species, mapping host associations onto a robust phylogeny can reveal whether ecological traits correspond to evolutionary lineages and help explain patterns of diversification.

In addition, Drepanosiphinae species are potentially characterized by atypical dual nutritional symbioses [14]. While most aphid species are associated with a single obligate symbiont, *Buchnera aphidicola*, which supplies essential amino acids and vitamin B that are limited or absent in the aphid diet, several studies using microbial metagenomic data have shown that this mutualistic relationship is not as exclusive as previously thought across the aphid phylogeny [14–16]. In several aphid subfamilies, *Buchnera* lacks essential metabolic functions, most notably the ability to synthesize biotin and riboflavin, and is complemented by other microbial symbionts that rescue or replace one or more of its roles. By compensating for the evolved auxotrophies of their *Buchnera* partners, these secondary symbionts provide essential nutrients and become co-obligate associates [14]. Such dual nutritional symbioses, in which *Buchnera* is complemented by an additional symbiont, have also been identified in members of the subfamily Drepanosiphinae. Notably, Fukatsu [17] discovered a secondary intracellular symbiotic bacterium in the genus *Yamatocallis* (in *Y. tokyoensis* Takahashi, 1923 and *Y. hirayamae* Matsumura, 1917), referred to as YSMS (*Yamatocallis* Secondary Mycetocyte Symbiont). Phylogenetically affiliated with the γ-Proteobacteria, this symbiont’s patterns of association and tissue tropism suggest a potential role in aphid nutrition. More recently, Manzano-Marín et al. [14] uncovered co-obligate associations involving *Sodalis*-like bacteria in other Drepanosiphinae members, i.e. *Drepanosiphum platanoidis* Schrank, 1801 and *Drepanosiphoniella fugans* Remaudière and Leclant, 1972. Genomic data from this study show that *Sodalis*-like symbionts, characterized by highly reduced genomes indicative of long-term obligate associations, appear to compensate for metabolic deficiencies in *B. aphidicola*, particularly the loss of B-vitamin biosynthesis. Although only four of the 42 recognized Drepanosiphinae species have been investigated for the presence of symbionts, the subfamily may represent an alternative example of a diversified obligate dual-symbiotic system, comparable to that observed in Chaitophorinae, Hormaphidinae or Lachninae [18, 19]. In order to investigate this hypothesis, a more thorough exploration of symbiont diversity across and within species is needed. A comprehensive phylogenetic framework will then help determining whether *Sodalis* is involved in a long-term association with this group and whether YSMS is restricted to certain species or genera.

In light of Drepanosiphinae’s strong host specificity, morphological complexity, and potential dual symbiotic systems, the overarching aim of this study is to provide the first comprehensive evolutionary framework for the group by integrating multilocus molecular phylogeny with endosymbiont characterization. Specifically, we aim to: 1. Reconstruct the phylogeny of 20 Drepanosiphinae species, representing five of the six recognized genera and nearly half of all known species; 2. Clarify the phylogenetic positions of the Asiatic genera *Yamatocallis* and *Megalosiphonaphis*, which exhibit unique morphological traits and restricted distributions; 3. Resolve relationships within *Drepanaphis*, the largest and most problematic genus, and assess whether the phylogenetic clustering of species reflects their host–plant associations with *Acer*; 4. Characterize endosymbiotic consortia across Drepanosiphinae, and map their distributions onto the host phylogeny in order to identify potential co-obligate symbionts.

## Material and methods

### Sample collection, fixation and storage

The study used 20 of 42 species from the subfamily Drepanosiphinae. Eight species belonging to the genus *Drepanaphis* [*D. acerifoliae*; *D. carolinensis* Smith, 1941; *D. granovskyi* Smith & Knowlton, 1943; *D. kanzensis* Smith, 1941; *D. monelli* (Davis, 1909); *D. robinsoni* Malik, 2024; *D. sabrinae* Miller, 1937; *D. utahensis* Smith & Knowlton, 1943] were collected in USA from 2022 to 2023, *D. acerifoliae* was also collected in Belgrade, Serbia in 2023. *D. simpsoni* Smith, 1959, collected in USA in 1976, was obtained from the MNHN collection (Paris, France). Four species belonging to the genus *Yamatocallis* [*Y. acericola* Higuchi, 1975; *Y. hirayamae*; *Y. nikkoensis* Sugimoto, 2017; *Y. tokyoensis*] were collected in Japan in 2023. *Megalosiphonapis nigrostriata* Sugimoto, 2024 was collected in Japan in 2024. *Drepanosiphoniella fugans* and *Drepanosiphum platanoidis* were collected in France in 2016. For each sample collected, a preliminary classification of individuals was carried out through observation under the Nikon SMZ 25 stereoscopic microscope. Subsequently, some specimens were mounted on slides and identified using a Nikon Ni-U light microscope equipped with a phase contrast system. All voucher specimens were preserved in 70% or 99.8% ethanol, stored at −20◦C and deposited in the entomological collection of the University of Silesia in Katowice, Poland (DZUS) and INRAe, Montpellier, France. Detailed collection data for all studied species are presented in Table S1.

## DNA extraction, polymerase chain reaction amplification and sequencing

For each collected aphid individual, total genomic DNA was conducted with a non-destructive DNA extraction as advised by [20]. We used the DNeasy Blood & Tissue Kit (Qiagen, Germany) and followed the manufacturer’s recommendations, except that that samples were left in the lysis buffer overnight and the final elution volume was 80 µl. During the extraction procedure, a negative control (i.e. a ‘blank template’ of ultrapure water) was processed with the same extraction kit. All DNA samples were stored at −20 °C. Polymerase chain reaction (PCR) amplifications were conducted for two mitochondrial gene fragments: a 700-bp region of the cytochrome oxidase I (COI), using primer pairs LepF (5’-ATTCAACCAATCATAAAGATATTGG-3’) LepR (5’-TAAACTTCTGGATGTCCAAAAAATCA-3’) [21] and 780 bp of the cytochrome b (Cytb) using primer pair CB1 (5’-GATGATGAAATTTTGGATC-3’) and CB2 (5’-ATTACACCTCCTAATTTATTAGGAAT −3’) [22]. We also amplified two nuclear gene fragments: 900 bp of the elongation factor-1a (EF1a) using primer pair EF3 (5’-GAACGTGAACGTGGTATCAC −3’) and EF6 (5’-TGACCAGGGTGGTTCAATAC-3’) [23] and 700 bp of the 6-phosphogluconate dehydrogenase PGD using primer pair PGD531 (5’-GGTGCTGGYCATTTYGTDAAAATG −3’) and PGD1097 (5’-CAKCCTCCACGCCACATDAG −3’) [24].

The PCR mixture included 12.5 μL of Master Mix with, 1.25 μL of each primer (10 μM), 7 μL of nuclease-free water and 2 μL of DNA template. Amplification included 30 s denaturation at 98 °C followed by 35 cycles each consisting of 30 s denaturation at 98 °C, 30s of annealing at temperature between 48 and 50°C°C and 1 min extension at 72 °C. A final extension was carried out at 72 °C for 5 min. PCR products were electrophoresed on 1% agarose to check for PCR success. Resulting PCR products were processed by the Eurofins sequencing society (Germany) using a BigDye v3.1 sequencing kit and Applied Biosystems 3730xl sequencers. Both strands were sequenced for all specimens to minimize PCR artefacts and ambiguities. Sequences of complementary strands were edited and reconciled using Geneious v11 (available at: http://www.geneious.com/).

Additional sequences were downloaded from GenBank (http://www.ncbi.nlm.nih.gov/Genbank): *Drepanaphis parva* Smith, 1941 (the only *Drepanaphis* available in GenBank prior to our study); *Drepanosiphum oregonense* Granovsky, 1939; *Yamatocallis sauteri* Takahashi, 1927. Two closely related Chaitophorinae species - *Chaitophorus salicti* Schrank, 1801 and *Periphyllus testudinaceus* (Fernie, 1852) were used as outgroup. In addition, we used a more distantly related Aphidinae species - *Brachycaudus helichrysi* (Kaltenbach, 1843) as an outgroup. GenBank sequence numbers for all studied species, including newly generated are presented in Table S1.

## Phylogenetic analyses

We aligned each gene fragment using ClustalW in MEGA v11 [25]. Sequences were then translated to check for any frameshift or stop codon. Then the two mitochondrial genes were concatenated in one alignment, and the nuclear gene fragments were concatenated in another alignment. Each of this dataset was analyzed separately to check for convergence of phylogenetic signal between maternally inherited DNA (mitochondrial data) and biparentally inherited data (nuclear DNA data). We then analyzed a complete data partition made of the concatenation of mitochondrial and nuclear genes.

For all DNA datasets we followed the same analytical protocol. In order to choose the most appropriate partitioning schemes and best substitution models, for each DNA matrix, we built a pre-partition file: we divided each protein–coding sequence into three partitions one for each codon position. We then ran ModelFinder as implemented in IQ-TREE v2.1.3 [26] with option MFP+MERGE which both tests, models for each DNA partition and test if partitions can be merged. Once models and partitions were selected, we ran Maximum Likelihood (ML) analyses in IQ-TREE v2.1.3 with 1000 ultrafast bootstrap replicates (-bb option), using the best fitting models and partitions found based on BIC (Bayesian information criterion) model selection.

Bayesian phylogenetic inference (BI) was further conducted using MrBayes v3.2. [27]. We partitioned the dataset by codon position, defining three partitions corresponding to the first, second, and third codon positions for mitochondrial genes and a single partition for the nuclear genes. For each partition, we applied a GTR substitution model with gamma-distributed rate variation among sites. Model parameters, including state frequencies, substitution rates, and gamma shape parameters, were unlinked across partitions to allow independent estimation. The analysis was run with four independent Markov chain Monte Carlo (MCMC) chains for 100 million generations, sampling every 1,000 generations. A temperature parameter of 0.07 was used to improve mixing. Convergence and stationarity were assessed by monitoring the standard deviation of split frequencies and effective sample sizes. The first 25% of samples were discarded as burn-in before summarizing parameter estimates and the posterior tree distribution.

## 16S rDNA endosymbiont characterization

Using the same DNA extractions as for aphid DNA sequencing, we amplified a 251-bp portion of the V4 region of the 16S rRNA gene [28] and used targeted sequencing of indexed bacterial fragments on a MiSeq (Illumina) platform using the dual-index sequencing strategy developed by [29], following the protocol described in Jousselin et al. [30]. DNA extracts were amplified twice along with negative controls. PCR replicates were conducted on distinct 96-well microplates.

The PCR products were pooled with samples from other microbiome studies, purified and quantified with the Kapa Library Quantification Kit (Kapa Biosystems). The DNA pool was then paired-end sequenced on an Illumina MiSeq flow cell with a 500-cycle Reagent Kit v2 (Illumina). We then followed the procedure of Manzano-Marín et al. [14] to analyze sequencing results and infer presence and absence of aphid endosymbionts. Briefly, sequencing results were first filtered through Illumina’s quality control procedure. We then used FLASH v1.2.11 [31] to merge paired sequences into contigs and CUTADAPT v1.9.1 to trim primers. The FROGS pipeline [32] was then used to generate an abundance table of symbiont lineages across samples. Taxonomic assignments of clusters was carried out using RDPtools v2.0.3 3(https://github.com/rdpstaff-/RDPTools, last accessed March, 2025 [33];) and, BLASTn+ against the Silva database release 138 [34] as implemented in FROGS. Following taxonomic affiliation, we aggregated clusters when they shared the same taxonomy with at least 98% of identity (FROGS’ affiliation postprocess step). We refined taxonomic assignation using phylogenetic placement of clusters using reference sequences of known facultative and obligate symbionts of aphids identified through genomic studies (Fig. S1). From the abundance table of clusters across samples, we transformed read numbers per aphid samples into percentages and sequences accounting for *<*0.5% of all the reads for a given sample were excluded using an R script following Jousselin et al. [30]. Clusters were kept only if present in both PCR replicates of the same sample. For final description of endosymbiont diversity, we only kept the PCR replicate that yielded the highest number of reads. We used relative abundance data to produce a heatmap in R of endosymbiotic taxa across aphid samples.

## Results

### Phylogenetic reconstructions within the subfamily Drepanosiphinae

**Fig. 1:**
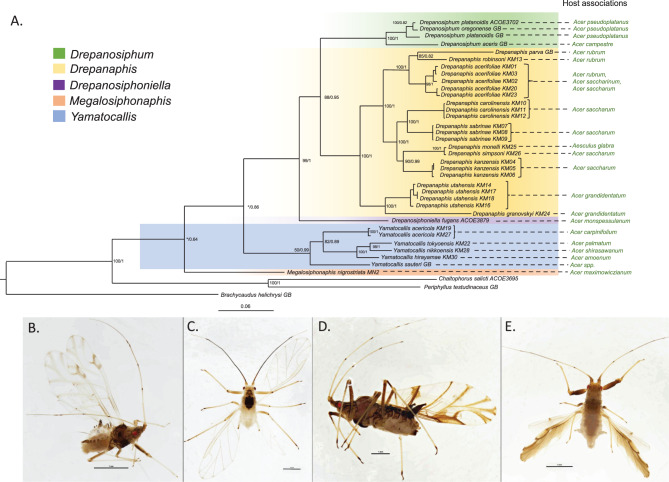
(**A**). Phylogenetic tree obtained from the combined analysis of mitochondrial and nuclear genes, with indication of host associations of analyzed aphid species of the Drepanosiphinae subfamily. The topology shown represents the tree inferred under Bayesian analyses based on four molecular markers: a 700-bp region of cytochrome oxidase I (COI), 780 bp of cytochrome b (cytb), 900 bp of elongation factor-1α (EF1α), and 700 bp of 6-phosphogluconate dehydrogenase (PGD). Values at nodes indicate bootstrap support from maximum likelihood (ML) analyses and posterior probabilities from Bayesian inference (BI) for congruent nodes. An asterisk (‘*’) indicates that the corresponding node was not recovered in the other analysis. Visual representation of species studied: fluid-preserved specimen of (**B**). *Drepanaphis acerifoliae*; (**C**). *Drepanosiphum oregonense*; (**D**). *Megalosiphonaphis nigrostriata*; (**E**). *Yamatocallis tokyoensis*

Phylogenetic analyses conducted using both ML and BI methods on a combined dataset (COI, Cytb, EF-1α and PGD genes) resulted in a largely similar topology within the ingroup (Fig. 1A). The Drepanosiphinae was found to be sister to the Chaitophorinae subfamily and retrieved as monophyletic but with quite low support (bootstrap support, BS = 55 posterior probability, PP = 0.64). The Drepanosiphinae representatives were split into two clades. One clade included all species of the monophyletic *Drepanaphis* (Fig. 1B) and representative species of *Drepanosiphum* (Fig. 1C) and *Drepanosiphoniella*, as sister groups to *Drepanaphis*. A second clade included all species of *Yamatocallis*. The genus *Megalosiphonaphis* (Fig. 1D) was either placed as a sister lineage of *Yamatocallis* (Fig. 1E) (in ML analysis) or sister to all other Drepanosiphinae (in the Bayesian inference).

The genus *Drepanaphis* was recovered as monophyletic with strong support (bootstrap support, BS = 100; posterior probability, PP = 1). Three clades included the following species of *Drepanaphis*: i) *D. granovskyi* and *D. utahensis* ii) *D. acerifoliae*, *D. parva*, *D. robinsoni* iii) *D. kanzensis*, *D. monelli, D. simpsoni*, *D. carolinensis* and *D. sabrinae*. The species belonging to the genus *Drepanosiphum* with *D. aceris*, *D, oregonense* and *D. platanoidis* formed a monophyletic group (bootstrap support, BS = 100; posterior probability, PP = 1). However, the analyzed *D. platanoidis* sequences come from distant populations that exhibit genetic variability. The genus *Yamatocallis* was recovered as monophyletic (BS = 82 PP = 0.99). *Y. tokyoensis*, *Y. nikkoensis* and *Y. hirayamae* constitute one clade, sister to species *Y. acericola* and *Y. sauteri*. As mentioned above, the position of *Megalosiphonaphis nigrostriata* varied and was poorly supported: it was found as a sister lineage to *Yamatocallis* (BS = 51) or sister to all other Drepanosiphinae (PP = 0.64).

Indicating host plant associations for each aphid species on the phylogenetic tree based on combined markers reveals that closely related species often utilize the same host plant (Fig. 1A). For example, *Drepanaphis utahensis* and *D. granovskyi*, which are sister species, are both associated with *Acer grandidentatum*. Overall, the distribution of host plant usage across the Drepanosiphinae phylogeny suggests that species feeding on similar hosts tend to be more closely related.

**Fig. 2:**
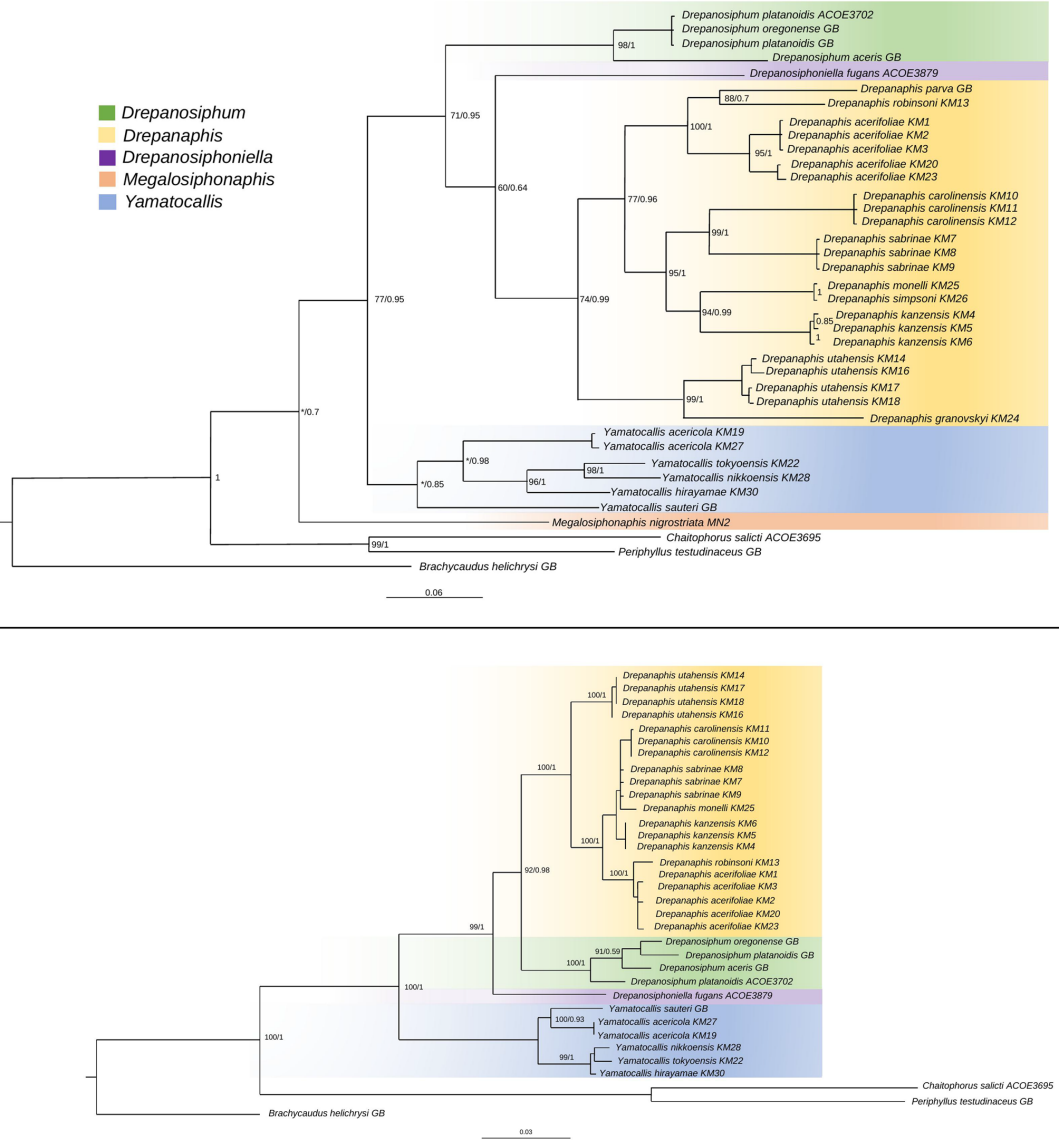
A. Phylogenetic tree obtained under Bayesian inference of mitochondrial markers: a 700-bp region of cytochrome oxidase I (COI), 780 bp of cytochrome b (cytb) of analyzed aphid species of the Drepanosiphinae subfamily. Values at nodes indicate bootstrap support (ML) and posterior probabilities (Bayesian inference) for congruent nodes. An asterisk (‘*’) indicates that the node was not recovered in the corresponding analysis. B. Phylogenetic tree obtained under Bayesian inference of nuclear markers: 900 bp of elongation factor-1α (EF1α), and 700 bp of 6-phosphogluconate dehydrogenase (PGD) of analyzed aphid species of the Drepanosiphinae subfamily. Values at nodes indicate bootstrap values obtained with posterior probabilities (Bayesian inference)

Analysis of mitochondrial genes (COI, Cytb) retrieved a tree (Fig. 2A) that was broadly congruent with the topology obtained from the full set of molecular markers, except for the placement of *Drepanosiphoniella fugans* (sister species to *Drepanaphis* in the mitochondrial tree but with low support: BS = 60 PP = 0.64). Nuclear genes (EF-1α, PGD) were sequenced for fewer taxa than mitochondrial ones and no sequences were obtained from the species: *D. granovskyi, D. parva*; *D. simpsoni* and *M. nigrostriata*. However, based on this limited sampling, our EF-1α and PGD tree (Fig. 2B) confirmed the monophyly of Drepanosiphinae (BS: 100; PP: 1). Similarly to the mitochondrial tree, one clade included genera *Drepanaphis, Drepanosiphum* and *Drepanosiphoniella*, but the position of *D. fugans* was different (sister to all others with strong support (BS: 100, PP: 1). *Yamatocallis* was found as a sister group to the remaining Drepanosiphinae.

There are differences in species groupings within the genus *Drepanaphis*. Phylogenetic analysis performed on nuclear markers clustered *D. monelli* between *D. carolinensis* and *D. sabrinae* which constitute a sister clade to *D. kanzensis*. Phylogenetic analyses performed on the combined data, groups *D. monelli* as a sister branch to *D. kanzensis*, constituting a sister clade to *D. carolinensis* and *D. sabrinae*.

## Diversity of symbionts associated with Drepanosiphinae

**Fig. 3:**
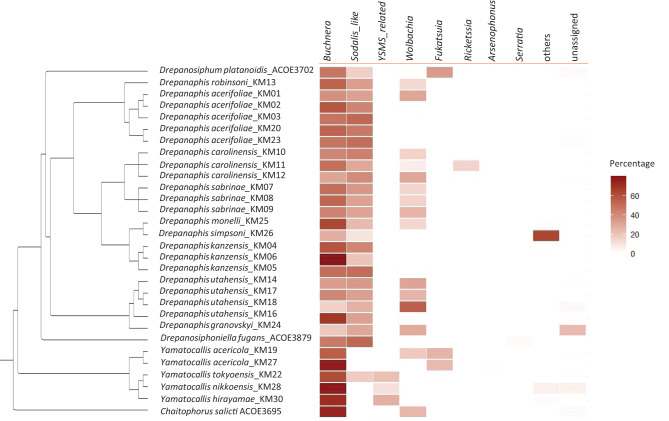
Phylogenetic tree and heatmap illustrating the distribution of endosymbionts among analyzed aphid species of the Drepanosiphinae subfamily. The tree topology used is the tree obtained with Bayesian analyses of combined markers. The heatmap (right) shows the relative 16S rDNA gene read abundance (in percentage) of different bacteria, identified as typical aphid endosymbionts (columns), detected in each species (rows). Gradient fill represents endosymbiont read relative abundance (in percentages), with darker colors indicating higher proportions. “Others” generally correspond to diverse bacteria that are ubiquitous and could represent environmental contaminations (all reads were added together for representation). “Unassigned” represent reads assigned to clusters that were in very low abundance (less than 0.5% of the reads found in a sample). The results are further detailed in additional files - Tables S1 and S2

High-throughput sequencing of 16s rDNA bacteria gene fragment yielded on average 15216 sequencing reads per sample, those were 251 bp long. This allowed uncovering the diversity of the aphid microbiota (Fig. 3; Table S2). In our 29 specimens from 16 species (*Drepanaphis acerifoliae*; *D. carolinensis*; *D. granovskyi*; *D. kanzensis*; *D. monelli*; *D. robinsoni*; *D. sabrinae*; *D. simpsoni*; *D. utahensis*; *Drepanosiphum platanoidis*; *Drepanosiphoniella fugans*; *Yamatocallis acericola*, *Y. hirayamae*; *Y. nikkoensis*; *Y. tokyoensis, Chaitophorus salicti*), 96 to 99% of the reads were assigned to the primary symbiotic partner of aphids, *Buchnera aphidicola* and several known aphid facultative symbionts: mainly a *Sodalis*-like bacterium, *Wolbachia*, *Ricketssia*, *Fukatsuia* and *Arsenophonus*. Using sequences from NCBI previously analyzed in Fukatsu [17], we also retrieved a cluster that was assigned to the so-called YSMS based on BLAST and phylogenetic analyses (Fig. S1, Table S3). Although results are represented as relative read abundances in the heatmap (Fig. 3), these values should not be interpreted as accurate estimates of symbiont abundance within specimens [30]. We therefore report and discuss only the presence or absence of symbionts. The obligate symbiont *Buchnera aphidicola* was consistently present across all species and individuals, whereas the presence of other symbionts varied among species and populations. A *Sodalis*-like symbiont was detected in 25 individuals, from Drepanosiphinae including all 22 individuals from *Drepanaphis* (*D. acerifoliae, D. carolinensis, D. granovskyi, D. kanzensis, D. monelli, D. robinsoni, D. sabrinae, D. simpsoni, D. utahensis*), the representative of *Drepanosiphoniella fugans*, *Drepanosiphum platanoidis* included here and a single individual from the *Yamatocallis* genus among the five included in our survey, namely *Yamatocallis tokyoensis*. Several sequence clusters had showed high sequence similarity with *Sodalis*-like symbionts previously identified in *Drepanosiphum platanoidis* [14] or Lachninae aphids [19] (Fig. S1, Table S3). Each aphid species always hosted a single *Sodalis* cluster, which suggests some kind of host specificity in *Sodalis*. Three *Yamatocallis* species, *Y. nikkoensis*, *Y. hirayamae* and *Y. tokyoensis* were found associated with a bacterial strain that showed genetic similarity with the YSMS, sequenced by Fukatsu [17]. This strain was not diversified across species, as it was represented by single genetic cluster in our sampling (Table S3, Fig S1). A more robust taxonomic affiliation of this strain would necessitate sequencing more genes and not just a short 16S rDNA gene fragment. Indeed, for instance, based on this fragment alone, *Sodalis* is not a monophyletic genus (Fig. S1). In any case, the heatmap of symbiont occurrence puts into phylogenetic perspective suggests acquisition of *Sodalis*-like in a common ancestor of *Drepanaphis*, *Drepanosiphoniella* and *Drepanosiphum* genera. *Wolbachia* strains are also largely distributed across Drepanosiphinae species: *Wolbachia* was detected in 14 individuals, including 13 individuals of *Drepanaphis* (*D. acerifoliae, D. carolinensis, D. granovskyi, D. monelli, D. robinsoni, D. sabrinae, D. utahensis)* and a single individual of *Yamatocallis acericola*. *Fukatsuia* endosymbionts was detected in both individuals of *Y. acericola* and *Drepanosiphum platanoidis. Ricketssia*, *Arsenophonu*s and *Serratia symbiotica* were all detected in a single individual from our sampling. A comparison of geographically distant populations from the native and introduced ranges revealed no differences in the composition of *Drepanaphis acerifoliae* populations from the USA and Europe. The complete composition of the microbiota (in relative read abundance) is available in Tables S2 and S3.

## Discussion

### Phylogenetic relationships within the subfamily Drepanosiphinae

Our phylogenetic analyses confirm the monophyly of Drepanosiphinae and its sister relationship to Chaitophorinae, consistent with the molecular framework proposed by Wieczorek et al. [1]. This result reinforces the monophyly of currently established subfamilies (sensu Remaudière and Remaudière) within Aphididae [24]. In contrast to the earlier study by Wieczorek et al. [1], which included only single mitochondrial or nuclear genes (COI and EF-1α) and a limited number of species, our research analyzed four gene regions and incorporated 20 species from five of the six recognized Drepanosiphinae genera (the lacking *Shenahweum* is a monotypic genus rarely collected in its Nearctic range). This broader dataset allowed for more robust resolution of internal relationships within the subfamily. The position of *Yamatocallis,* in particular has been clarified. While previously represented only by *Y. tokyoensis* and considered an independent lineage within Drepanosiphinae, our study included four additional species (*Y. acericola, Y. nikkoensis, Y. hirayamae*, and *Y. sauteri*). The results strongly support *Yamatocallis* as a sister clade to the main Drepanosiphinae lineages, which includes *Drepanaphis*, *Drepanosiphum* and *Drepanosiphoniella*. This finding aligns with analyses of morphological features and supports the inclusion of *Yamatocallis* within the subfamily, rather than as a separate lineage. The newly described monotypic genus *Megalosiphonaphis* [11], was retrieved as part of the Drepanosiphinae clade, but with low branch support (below 0.7). This uncertainty is likely due to the limited data available, as only mitochondrial markers were successfully sequenced for this genus. Morphologically, *Megalosiphonaphis* shares traits with other Drepanosiphinae, such as elongated siphunculi with a reticulated apex (similar to *Yamatocallis*) and the arrangement of accessory rhinaria on antennal segment VI (as in *Drepanosiphum*), suggesting a close relationship. However, more comprehensive molecular data are needed to confirm its systematic placement. Our results also support the monophyly of *Drepanosiphum*. However, two populations of *D. platanoidis* were placed in separate positions on the phylogenetic tree, likely reflecting intraspecific genetic variability rather than species-level divergence. The genus *Drepanosiphoniella* was also recovered within the core Drepanosiphinae clade, confirming its close relationship with *Drepanosiphum* and *Drepanaphis*, as previously suggested [1]. These findings emphasize the importance of combining broader taxon sampling with multilocus molecular data to refine the systematics within aphid subfamilies and to resolve longstanding taxonomic uncertainties within the group. Thorough molecular phylogenetic investigations have been conducted for several aphid subfamilies (Calaphidinae [35]; Chaitophorinae [36]; Lachninae [37]), but many aphid lineages still lack such analyses. Thus, we provide here the most comprehensive phylogenetic reconstruction to date for Drepanosiphinae.

Morphological affinities, versus phylogenetic relationships in *Drepanaphis* with insight into their biogeographical history and host-plant associations

*Drepanaphis*, is the most diverse genus within the subfamily Drepanosiphinae, as it includes 18 species [9]. Winged viviparous females are distinguished by the tubercles on the dorsal part of the abdomen, which are the most remarkable features in this group. Over the last decades, the most reliable source of information about the *Drepanaphis* species has been the revision conducted by Smith and Dillery [13]. Although they did not incorporate all critical diagnostic features of the winged viviparous females necessary for determining morpho-groups, the revision also overlooked the descriptions of all known oviparous females and males. Recently, Malik et al. [9] conducted a comprehensive morphological revision of *Drepanaphis*, using a matrix of 52 characters and broad comparative material. This study clarified previously problematic taxa such as *D. nigricans* Smith, 1941 and *D. tissoti* Smith, 1944, enabled the identification of all morphs, and resulted in the delineation of five morpho-groups: “acerifoliae,” “monelli,” “nigricans,” “parva,” and “utahensis”. Present molecular phylogenetic analyses partly supported the morphological groupings but also revealed some inconsistencies. Only 10 of the 18 known species of *Drepanaphis* were available for molecular study, leaving some groups - such as “nigricans” - unrepresented. Phylogenetic trees based on mitochondrial and nuclear markers were mostly congruent, with the exception of a few taxa. Species in the “acerifoliae” group, though morphologically similar, did not form a monophyletic clade. Instead, *D. acerifoliae*, *D. parva*, and *D. robinsoni* appeared in closely related yet distinct lineages. All three species are associated with *Acer rubrum*, and while *D. parva* and *D. robinsoni* were previously grouped morphologically, molecular results indicate they form a sister clade to *D. acerifoliae*, an oligophagous species on the same host. This clustering of species using the same range of host-plants, suggests that host plant specialization triggers speciation events in this aphid group [38]. Host plant associations also help clarify relationships in the “utahensis” group. *D. granovskyi* and *D. utahensis*, both monophagous on *Acer grandidentatum* and restricted to the western United States, form a distinct clade. In contrast, *D. simpsoni*, also in the “utahensis” morpho-group and associated with *Acer saccharum*, belongs to a separate, distributed in the eastern United States lineage. These phylogenetic splits likely reflect both host specificity and geographic isolation as triggering factor of speciation events as observed in other Nearctic aphids [39, 40]. Notably, *D. monelli*, the only species in the genus associated with *Aesculus glabra* rather than maples, showed inconsistent phylogenetic placement. Mitochondrial data clustered it with *D. simpsoni*, forming a sister group to *D. kanzensis*, while nuclear data placed it with *D. sabrinae* and *D. carolinensis*. The lack of nuclear data for *D. simpsoni* may explain this incongruence. Morphologically, *D. monelli* has been linked to species such as *D. keshenae*, *D. knowltoni*, and *D. spicata*, which were not included in the molecular dataset due to limited material. Some species not assigned to any morphological group, like *D. sabrinae* and *D. kanzensis* are clustered with species sharing the same host plant in molecular analyses, further supporting the value of host association as a taxonomic criterion. Additionally, several monophagous species that are grouped together phylogenetically also share morphological traits, suggesting convergence under similar ecological pressures. Thus, based on the combined morphological, molecular, and ecological evidence, we propose a revised species grouping system for *Drepanaphis*, centered on host plant affiliation. Three major host-associated groups are recognized: the *rubrum*, *saccharum*, and *grandidentatum*. This host-based classification more accurately reflects the evolutionary history and highlights the conserved nature of host plant associations in the genus *Drepanaphis*.

## Drepanosiphinae’s endosymbiotic consortia

Our results provide new insight into the diversity of microbial communities associated with Drepanosiphinae, highlighting the complexity and variability of endosymbiotic relationships in this aphid subfamily. As expected, the obligate endosymbiont *Buchnera aphidicola* was present in all individuals. However, significant variation was observed in the composition of secondary symbionts across species and populations. The dominant facultative symbiont identified was a *Sodalis*-like bacterium, detected in 86% of Drepanosiphinae individuals. It was present in all species of *Drepanaphis*, *Drepanosiphoniella*, and *Drepanosiphum*, and in all individuals sampled from these genera. Its occurrence did not vary with host plant association or geographic distribution. A single strain was found per aphid species which suggests some kind of host specificity in *Sodalis*. Previous genomic studies on *Drepanosiphum platanoidis* showed that its *B. aphidicola* lacks essential biosynthetic capacities, which appear to be complemented by a *Sodalis*-like symbiont that shows genomic characteristics typical of long-term obligate symbiosis (i.e., highly reduced genome and high GC content). These findings led to the suggestion that *Sodalis*-like bacteria may act as co-obligate partners of *Buchnera* in *D. platanoidis* and potentially in other Drepanosiphinae species [14].

The symbiont distribution patterns observed in our study support this hypothesis and suggest that the acquisition of a *Sodalis*-like symbiont occurred early in the evolutionary history of Drepanosiphinae. However, species of the genus *Yamatocallis* do not show a systematic association with a *Sodalis*-like partner. Fukatsu [17], based on limited sampling of *Y. tokyoensis* and *Y. hirayamae*, proposed that these species harbor a distinct intracellular symbiont, YSMS, suggesting an ancient association dating back to the origin of the genus. We detected YSMS-like sequences in three *Yamatocallis* species, but a robust taxonomic placement of this bacterium will require sequencing of additional genetic markers. Based solely on the 16S rRNA gene fragment, we cannot rule out the possibility that YSMS also belongs to the *Sodalis* lineage (Fig. S1). Regardless of its precise taxonomic status, we did not detect YSMS or any *Sodalis*-like symbiont in *Yamatocallis acericola*, which argues against Fukatsu’s hypothesis [17] and instead points to two alternative scenarios: a more recent acquisition of YSMS in a subset of species, or a lineage-specific secondary loss. A further possibility is that *Yamatocallis* initially harbored *Buchnera* together with *Fukatsuia*, and that *Fukatsuia* was later replaced by YSMS. Additional data, particularly from *Y. sauteri*, will be essential to discriminate among these hypotheses. Endosymbiont profiling using multiple markers or whole endosymbiont genome data across species are also needed to: 1) elucidate the taxonomic affiliation of YSMS; 2) reconstruct the phylogenetic history of *Drepanosiphinae* main symbionts (*Sodalis* and YSMS) and evaluate aphid/symbiont codiversification scenarios (as [41]). In addition to *Sodalis*, the facultative symbiont *Wolbachia* was detected in 48% of Drepanosiphinae individuals, a notably high prevalence compared to other aphids [42, 43]. However, unlike *Sodalis*, *Wolbachia* was not present in all individuals of a given species and thus cannot be considered an obligate partner. Understanding its ecological role in Drepanosiphinae will require further genomic and ecological research. Given aphids’ reproductive biology, it is unlikely that *Wolbachia* functions as a reproductive manipulator. Nonetheless, it has been shown to protect aphids from fungal pathogens in *Pentalonia nigronervosa* (Coquerel, 1859) [44]. Thus, Drepanosiphinae may provide a promising system for comparative studies of *Wolbachia* in aphids, given the relatively high and recurrent detection of this symbiont in multiple species. Unlike *Cinara cedri* Mimeur, 1936 [45] or *P. nigronervosa,* where infection can reach nearly 100% of samples, Drepanosiphinae species exhibit variable prevalence of *Wolbachia*, making them particularly valuable for studying the evolutionary dynamics and ecological correlates of symbiont maintenance and loss [30, 42].

Other symbionts, including *Fukatsuia*, *Rickettsia*, *Serratia*, and *Arsenophonus*, were found only in a few individuals, suggesting transient or facultative associations. These rare occurrences reflect the dynamic nature of aphid microbiomes, where facultative symbionts may be gained or lost in response to environmental pressures, host plant use, or other ecological factors [46, 47].

Overall, our findings emphasize the value of combining high-throughput sequencing with ecological and taxonomic context to better understand the evolution of symbiotic associations in aphids. In Drepanosiphinae, *Sodalis*-like bacteria potentially play a central role in compensating for *Buchnera* deficiencies. However, symbiont diversity differs markedly between lineages, as illustrated by the contrasting profiles of *Yamatocallis*. Similar differences in co-obligate associations have been observed in Chaitophorinae [14, 15, 48], the sister group to Drepanosiphinae, where *Serratia symbiotica* appears to have been independently gained or lost multiple times. Such evolutionary lability in nutritional symbioses was first highlighted in Lachninae [18, 19, 49]. The significant role of endosymbionts in various insect groups is supported not only by molecular studies but also by morphological analyses, revealing their diversity and intergenerational transmission strategies [50].

Thus, our results further highlight the diversity of nutritional symbiotic consortia in aphids and emphasize the potential of Drepanosiphinae as an alternative model system for exploring the evolution of dual symbiotic associations in aphids.

## Conclusions

This study provides a comprehensive phylogenetic framework for the subfamily Drepanosiphinae, confirming its monophyly and clarifying relationships among its genera. Phylogenetic analyses consistently support a close relationship between *Drepanaphis* and *Drepanosiphum*, with *Drepanosiphoniella* forming a sister lineage, while *Yamatocallis* and *Megalosiphonaphis* represent distinct evolutionary branches. Within *Drepanaphis*, host plant association emerges as a key factor shaping species groupings, leading to the definition of three host-associated groups: *rubrum*, *saccharum*, and *grandidentatum*, which correspond to specific *Acer* host affiliations. The widespread occurrence of a *Sodalis*-related bacterium across Drepanosiphinae, along with previous genomic evidence, suggests a long-standing co-obligate symbiosis within the subfamily. *Wolbachia* is also recurrently associated with members of the subfamily, contrasting with earlier reports of its low prevalence in aphids, and raising questions about its ecological role in this group. Additional facultative symbionts, including *Rickettsia*, *Fukatsuia*, *Serratia* and *Arsenophonus*, exhibit limited and sporadic distributions, possibly reflecting transient or non-established, rather than stable, long-term coevolutionary associations. Together, these findings significantly enhance our understanding of phylogenetic relationships within Drepanosiphinae and provide new insights into the diversity and evolutionary dynamics of aphid-symbiont interactions.

## Electronic supplementary material

Below is the link to the electronic supplementary material.

Supplementary Material 1

Supplementary Material 2

Supplementary Material 3

Supplementary Material 4

## Data Availability

All data generated or analyzed during this study are included in this published article [and its supplementary information files]. Genetic data are deposited in GenBank, NCBI.
