# Supplementary figures and images for: Molecular phylogeny of the Acer-feeding aphid subfamily Drepanosiphinae (Insecta: Hemiptera: Aphididae) and the evolution of its endosymbiotic consortia
Source: Zoological Lett. 2025 Dec 13;11:9. doi: 10.1186/s40851-025-00255-2 (PMC12709785; doi:10.1186/s40851-025-00255-2)

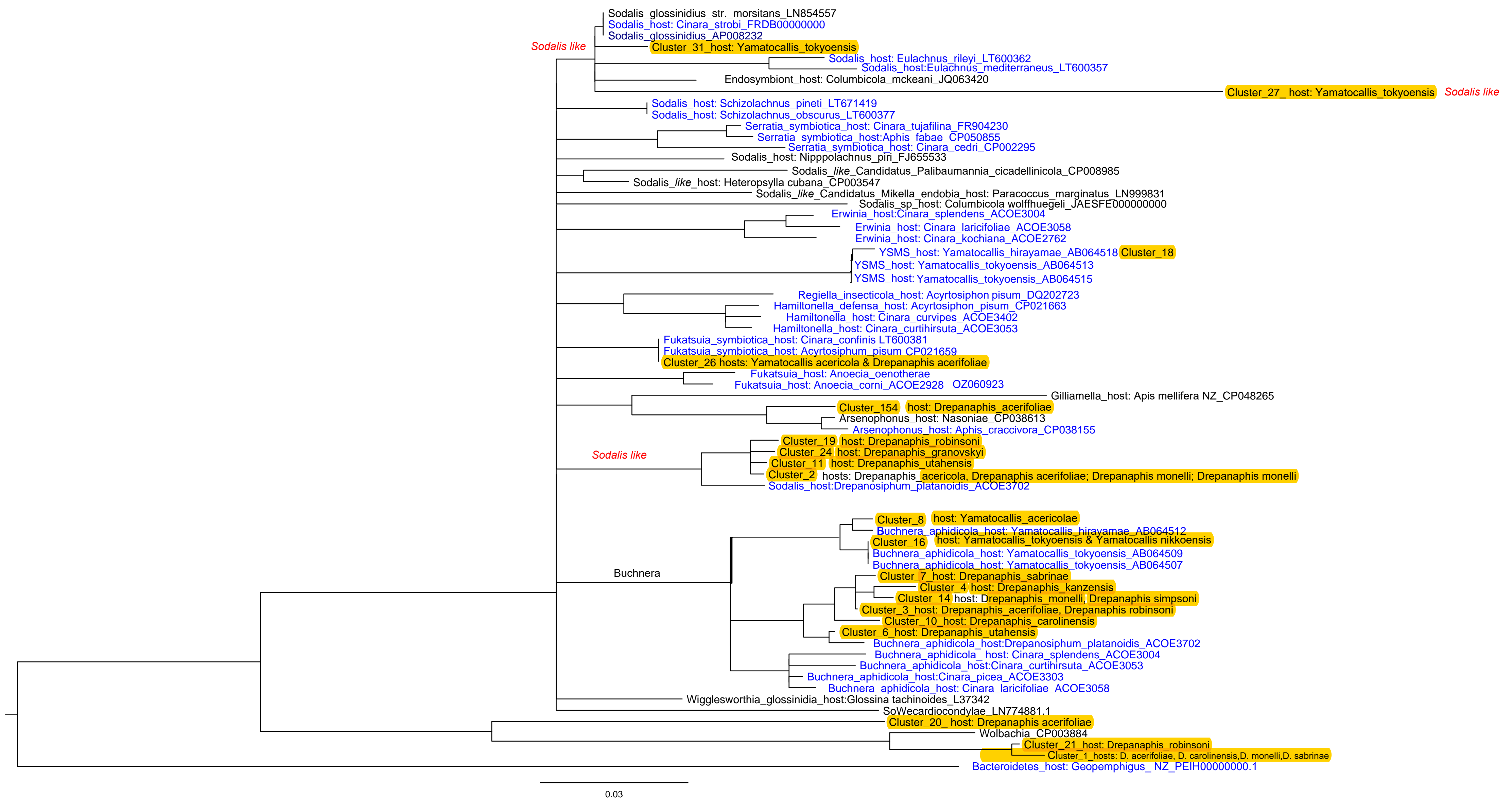

Supplement: Supplementary file 1 — Supplementary Material 1 [file 40851_2025_255_MOESM1_ESM.pdf]
